# miR-331-3p Inhibits Inflammatory Response after Intracerebral Hemorrhage by Directly Targeting NLRP6

**DOI:** 10.1155/2020/6182464

**Published:** 2020-06-07

**Authors:** Hao Nie, Yang Hu, Wenliang Guo, Wenzhi Wang, Qingwu Yang, Qiang Dong, Yuping Tang, Qi Li, Zhouping Tang

**Affiliations:** ^1^Department of Neurology, Tongji Hospital, Tongji Medical College, Huazhong University of Science and Technology, Wuhan Hubei 430030, China; ^2^Department of Geriatrics, Tongji Hospital, Tongji Medical College, Huazhong University of Science and Technology, Wuhan, Hubei 430030, China; ^3^Beijing Neurosurgical Institute, Tiantan Hospital, Capital Medical University, Beijing 100000, China; ^4^Department of Neurology, Xinqiao Hospital and The Second Affiliated Hospital, Army Medical University (Third Military Medical University), Chongqing 400037, China; ^5^Department of Neurology, Huashan Hospital, Fudan University, Shanghai, China; ^6^Department of Neurology, The First Affiliated Hospital of Chongqing Medical University, Chongqing 400037, China

## Abstract

**Background:**

The mechanism of inflammatory reaction after intracerebral hemorrhage remains unclear, which to some extent restrains the therapeutic development of hemorrhagic stroke. The present study attempts to verify whether NLRP6 plays an important role in inflammatory reaction after intracerebral hemorrhage and identify the critical microRNA during the process.

**Methods:**

Suitable simulated cerebral hemorrhage environments were established *in vitro* and *in vivo*. BV2 cells were treated with hemin to induce cell damage. Collagenase was used to establish a model of mouse cerebral hemorrhage. The relationship among NLRP6, miR-331-3p, and the corresponding inflammatory expression was closely observed during this process. Techniques, such as western blot, real-time quantitative PCR, immunofluorescence, and immunocytochemistry, were used to detect the expression of relative genes and molecules in the *in vitro* and *in vivo* models.

**Results:**

Downregulated miR-331-3p increased the expression of NLRP6 and alleviated the expression of TNF-*α* and IL-6. The neurological function recovery of mice was promoted after intracerebral hemorrhage.

**Conclusion:**

miR-331-3p regulated the inflammatory response after cerebral hemorrhage by negatively regulating the expression of NLRP6.

## 1. Introduction

Intracerebral hemorrhage (ICH) is a severe neurological disease. At present, the specific mechanism of inflammatory reaction after ICH remains unclear, and this has become a hotspot in the field of neuroscience in recent years.

Microglia are glial cells that damage neurons and have a similar phagocytosis to macrophages. Microglia activate inflammatory cells through the release of neurotoxic factors and proinflammatory factors, including tumor necrosis factor-*α* (TNF-*α*) and interleukin-6 (IL-6), thereby activating downstream signaling pathways and regulating inflammatory response [1, 2]. It has been proven that microglia respond very actively during ICH and can be considered as representative cells of inflammatory response in the brain after ICH [3].

The nucleotide-binding oligomerization domain, leucine-rich repeat, and pyrin domain-containing protein (NLRP) family has been reported to be involved in the immune response and plays an important role in apoptosis, inflammation, and immune response. NLRP6 is the first member of the NLRP family reported to inhibit innate immune responses. It is a negative regulator of inflammatory signaling and has a role in assisting the clearance of Gram-positive and Gram-negative bacterial pathogens [4]. Studies on NLRP6 in the field of neurosciences have mainly focused on peripheral nerves, which can promote the repair of peripheral nerve injury [5, 6].

MicroRNAs are noncoding RNAs with a length of 21-25 nucleotides. These can pair with the mRNA of the target gene, inhibit the translation process of mRNA, or cause the degradation of mRNA [7], thereby regulating the transcriptional expression of the target gene [8]. MicroRNAs respond in the early stage of the pathophysiological process. It has been reported that microRNAs play an important role in the diagnosis and treatment of diseases [9, 10].

The present study was aimed at assessing the immune-modulatory effects of NLRP6 and associated microRNAs on microglia after ICH *in vitro* and *in vivo*.

## 2. Materials and Methods

### 2.1. Ethics Statement

The experimental protocols were approved by the Regulations for the Administration of Affairs Concerning Experimental Animals drafted by the Chinese Ministry of Science and Technology. Male C57BL/6 mice (8-10 weeks old, weighing 22-26 g) were provided by the Animal Center of Tongji Medical College. These mice were settled in separate cages with free access to clean water and food.

### 2.2. Cell Culture

BV2 microglia were purchased from the American Type Culture Collection and cultured in T25 flasks using DMEM medium (Gibco, USA) containing 10% fetal bovine serum (Gibco, USA) at 37°C. The cell passages were performed when the cell density reached 80%-90% confluency.

### 2.3. Treatment with Hemin

Cells were seeded in a six-well plate or a culture flask at a cell density of 2 × 10^5^/mL. When the cell density reached 40%-50% confluency, hemin was added into a six-well plate at different concentrations and cultured for 24 hours. Then, these cells were harvested for the following detection.

### 2.4. Interference of BV2 with the miRNA Mimic and Inhibitor

BV2 microglia were cultured in a 6-well plate at a cell density of 2 × 10^5^/mL. When the cell density reached 40%-50% confluency, the miRNA-331-3p mimic, mimic negative control, inhibitor, and inhibitor negative control were transfected into the BV2 microglia at a concentration of 100 *μ*M using an Opti-MEM® I Reduced Serum Medium (Gibco, USA) and Lipofectamine 3000 (Invitrogen, USA). The Opti-MEM medium was replaced with a complete medium at six hours after transfection. After the transfection was completed, hemin was added into the six-well plate and cultured for 24 hours.

### 2.5. Dual Luciferase Reporter Assay

Compared with the negative control, the wild-type vector reported the downregulation of the fluorescence of the miRNA mimics, and the reporter fluorescence of the mutant vector was restored, suggesting that the miRNA mimics have a regulatory effect on the reporter gene. Then, 293T cells were seeded into 96-well plates at a density of 1.0 × 10^4^ cells per well. Afterwards, the miR-331-3p mimic or negative control sequences (RiboBio, China) and dual luciferase vector carrying the wild-type or mutant sequences were cotransfected. The sequences were as follows: NLRP6 WT, 5′-CCAGGGGA-3′, and NLRP6 MUT, 5′-GGTCCCCT-3′. The Dual-Glo® Luciferase Assay System (Promega, USA) was used to measure the luciferase activities of cells, according to the manufacturer's instructions. The Renilla-to-firefly luciferase signal ratio was used to normalize the luciferase activity. The interaction between the miRNA and target gene was confirmed by the downregulation of the relative fluorescence of the reporter gene.

### 2.6. Quantitative Real-Time Polymerase Chain Reaction (PCR)

Total RNA was extracted from the BV2 microglia and brain tissue using TRIzol (Invitrogen, USA), according to the manufacturer's instructions. The microRNA was extracted using a miRNA Isolation Kit with Spin Column (Beyotime, China). The RNA reverse transcription and real-time PCR were conducted using a PrimeScript™ RT Reagent Kit (TaKaRa, Japan) and TB Green® Premix Ex Taq II Kit (TaKaRa, Japan), according to the manufacturer's protocols (ABI StepOnePlus™, USA). The miRNA constructed the first-chain sequencing library using a Mir-X miRNA First-Strand Synthesis Kit (TaKaRa, Japan). The real-time PCR was performed using a Mir-X miRNA qRT-PCR TB Green® Kit (TaKaRa, Japan). The 2^*ΔΔ*Ct^ method was applied to compare the differences among the different samples. GAPDH and U6 were used as internal references for mRNA and miRNA, respectively.

### 2.7. Western Blot

Treated cells and brain tissue around the hematoma were harvested for western blot. RIPA lysis buffer and PMSF were applied to homogenize the collected sample. A BCA Protein Assay Kit (Beyotime, China) was used to measure the protein concentration. The operation steps of the western blot were the same as those previously described [11]. The membranes were incubated with anti-NLRP6 (1 : 1,000; Santa Cruz, USA) and anti-GAPDH (1 : 1,000; Abcam, UK) overnight. The bands were visualized using a Gel Doc® XR+ System (Bio-Rad, USA), and the density of the visualized bands were analyzed using ImageJ 1.50b (National Institutes of Health, USA).

### 2.8. Flow Cytometry Analysis of Cell Apoptosis

BV2 microglia were harvested using 0.05% trypsin solution without EDTA (Gibco, USA), washed with phosphate buffer saline solution (Biological Industries, Israel) for three times, and resuspended with a Binding Buffer (MultiSciences, China). Annexin V-fluorescein isothiocyanate (V-FITC) and propidium iodide (PI) were used for cell staining, according to the manufacturer's protocol. Apoptotic microglia (annexin V-FITC+/PI-) were detected using a flow cytometer (BD, USA).

### 2.9. Immunofluorescence Staining

BV2 microglia were washed with phosphate buffer saline solution; fixed with 4% buffered paraformaldehyde; incubated overnight at 4°C with primary antibodies, including anti-NLRP6 (1 : 500; Santa Cruz, USA) and anti-Iba-1(1 : 500; Novus, USA); and incubated with anti-goat secondary antibody (Proteintech, USA) and anti-rabbit secondary antibody (Proteintech, USA). Then, the cell nuclei were stained with DAPI (Beyotime, China). Cells were observed under an inverted fluorescence microscope (Olympus, Japan).

### 2.10. ICH Model

Collagenase type VII (Sigma, USA) was used to induce the mouse ICH model, and sterile saline was used as the vehicle control group. Male C57BL/6 mice (8-10 weeks old, weighing 22-26 g) were well-anesthetized and gently fixed in a stereotaxic instrument (RWD, China). The head of these mice was positioned parallel to the locator base, and the skin in the surgical area was disinfected. Coordinates at 0.2 mm anterior, 2.5 mm lateral, and 3.5 mm vertical to the bregma were located as the origin point. Collagenase (0.06 U per mouse) or sterile saline was slowly injected into the right basal ganglia using a microinjection pump. The operative incision was sutured. Then, the condition of the incision and the general behavior and symptoms of the mice were observed daily.

### 2.11. Laser Speckle Imaging

Cerebral blood flows were evaluated by laser speckle imaging. Mice were fully anesthetized and fixed. After the frontal bone and parietal bone were exposed, a groove was formed along the periphery of the cranial window. The laser speckle contrast imaging system (Perimed, Sweden) was used to monitor the blood flow, and the focal length of the stereo microscope was adjusted to find the best observation area. The cranial window was irradiated evenly with the laser. The cerebral cortex blood flow changes of mice were observed and collected. After the calculation of the algorithm, a two-dimensional distribution map of cerebral blood flow velocity is obtained.

### 2.12. miRNA Administration

Agomir-331-3 and Agomir negative control were dissolved in sterile saline and injected at a dose of 2 nmol per mouse after the collagenase. In addition, Agomir-331-3p and Agomir negative control were injected through the tail veins of the model mice every 12 hours at a dose of 3 nmol/10 g of body weight for three days. The remaining feeding environment and treatment methods were consistent. The brain samples were taken or perfused according to the experimental requirements, and the corresponding components were collected for detection at three days after administration.

### 2.13. Behavioral Assessments

Modified neurological severity scores (mNSS) were double-blindly used to assess the behavioral functions on days 0, 1, 3, 5, and 7 after surgery [12]. The test was graded on a scale of 0 (normal score) to 10 (maximum deficit score). Each mouse was tested for three times.

### 2.14. Immunocytochemistry

The mice were deeply anesthetized and perfused with 0.9% saline, followed by 4% paraformaldehyde. Whole brains were paraffin-embedded and sliced into 4 *μ*m thick tissue sections for immunohistochemical observation. The brain tissue sections were dewaxed with xylene, hydrated in graded ethanol, incubated with 3% hydrogen peroxide, and incubated overnight at 4°C with the primary antibody anti-NLRP6 (1 : 100; Santa Cruz, USA) and anti-goat secondary antibody (Proteintech, USA). Afterwards, the sections were added with droplets of DAB for incubation and counterstained with hematoxylin. All stained slides were viewed under a microscope (Olympus, Japan).

### 2.15. Statistical Analysis

Statistical analysis was performed using the SPSS Statistics 17.0 software. Data were expressed as mean ± standard deviation (SD). All data were analyzed using Student's *t*-test and one-way ANOVA, followed by the Tukey post hoc test. A *P* value of <0.05 was considered statistically significant.

## 3. Results

### 3.1. Hemin Induces the Immune Response of BV2 Cells

**Figure 1:**
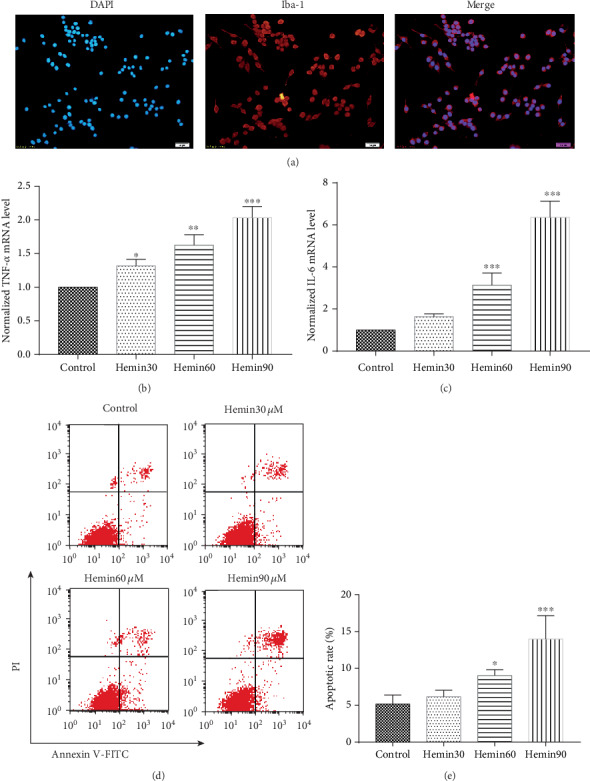


Hemoglobin decomposed after ICH and released a very large amount of hemin. The secondary brain injury was partly due to the toxic effect of hemin, which induced the increased expression of inflammatory factors and cell death in the periphery of the hemorrhagic foci [13–15]. BV2 microglia were used to explore the inflammatory effects after ICH in an *in vitro* model. The identification of BV2 cells is presented in Figure 1(a). TNF-*α* and IL-6 were chosen as indicators of inflammatory factors, and flow cytometry was performed to measure the BV2 cell apoptosis rate. As the concentration of hemin increased, the expression level of TNF-*α* and IL-6 in BV2 cells significantly increased (Figures 1(b) and 1(c)), while cell viability decreased (Figures 1(d) and 1(e)). Furthermore, 60 *μ*M was chosen as a reasonable concentration of hemin for the subsequent treatment of cells.

### 3.2. NLRP6 Expression Was Upregulated in Hemin-Treated BV2 Cells

**Figure 2:**
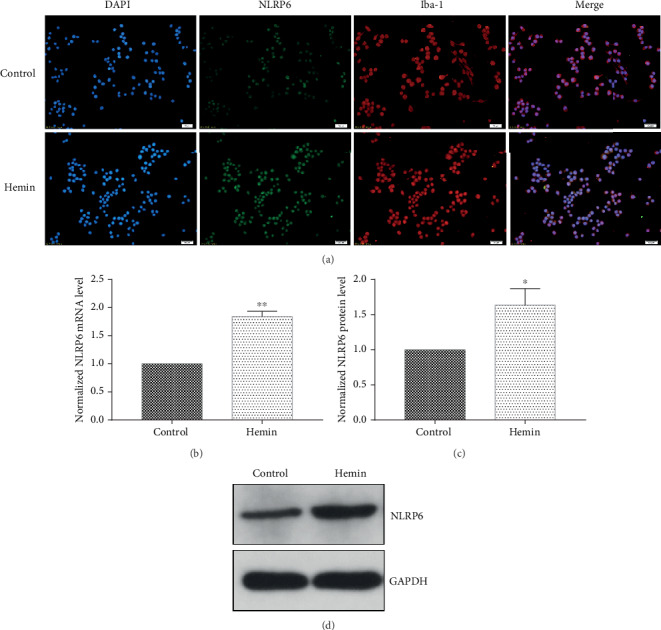


In order to verify the role of NLRP6 in hemin-treated BV2 cells, the expression of NLRP6 was determined by immunofluorescence, real-time quantitative PCR, and western blot assay. The results indicated that compared to the control group, NLRP6 exhibited stronger expression from the mRNA level to the protein level after hemin treatment (Figure 2).

### 3.3. miR-331-3p Expression Is Downregulated in Hemin-Treated BV2 Cells

**Figure 3:**
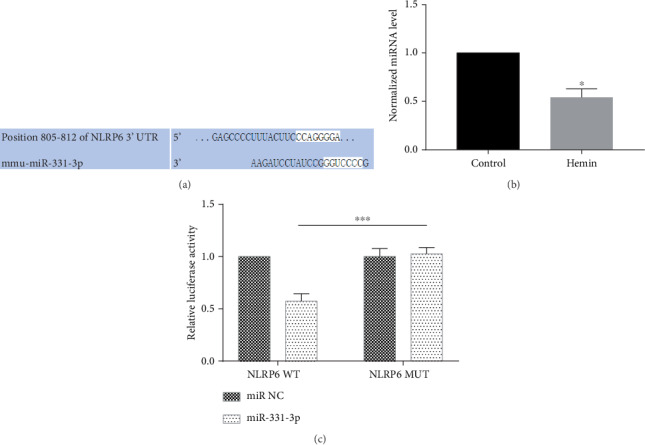


In order to further explore the potential regulatory mechanism, miR-331-3p was identified as the possible conserved target with the use of TargetScan (version 6.2) (Figure 3(a)). The real-time quantitative PCR confirmed that the expression of miR-331-3p in hemin-treated BV2 cells was significantly lower, when compared to the control group (Figure 3(b)). miRNA regulates the expression of target genes through the 3′UTR region. By cotransforming the miRNA with the constructed reporter gene vector and detecting whether the relative fluorescence of the reporter gene is downregulated, it can be verified whether the miRNA regulates the target gene. The dual luciferase reporter gene assay verified that miR-331-3p has a regulatory effect on NLRP6 (Figure 3(c)).

### 3.4. The Regulation of NLRP6 by miR-331-3p Influences the Inflammatory Response of Hemin-Treated BV2 Cells

**Figure 4:**
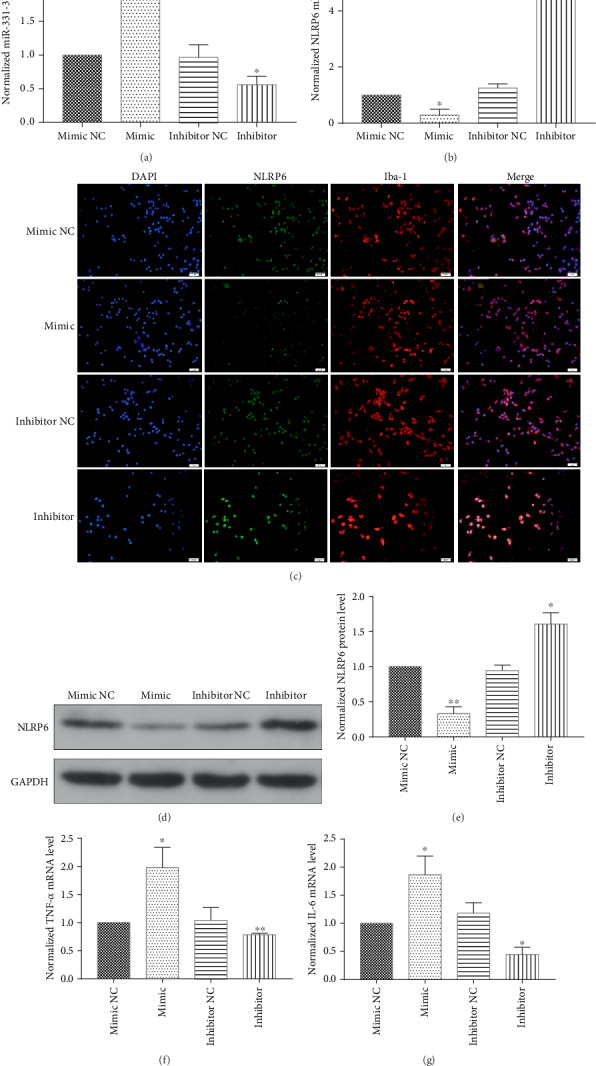


miRNA mimics and inhibitors were used to regulate the expression of miR-331-3p in the transfected BV2 microglia (Figure 4(a)). The real-time quantitative PCR, immunofluorescence, and western blot revealed that miR-331-3p negatively regulated the NLRP6 expression (Figures 4(b)–4(e)). In the meantime, in order to show that the miR-331-3p mimic and inhibitor regulate the immunosuppressive capacity of BV2 microglia, downstream inflammatory factors TNF-*α* and IL-6 were detected, and it was revealed that the downregulation of miR-331-3p inhibited the inflammation reaction of hemin-treated BV2 microglia and vice versa (Figures 4(f) and 4(g)).

### 3.5. miR-331-3p Was Downregulated and NLRP6 Expression Was Upregulated in the ICH Mouse Model

**Figure 5:**
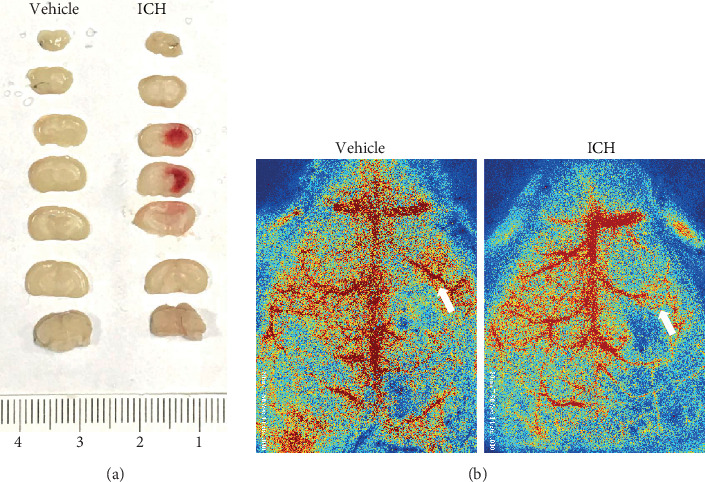


**Figure 6:**
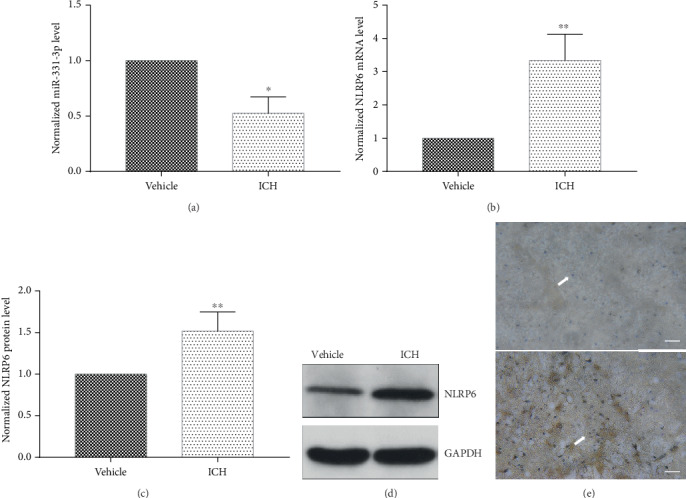


In order to verify whether the expression of miR-331-3p in mice after ICH is the same as the tendency in the cell experiments, brain tissues around the hematoma were collected and the expression of miR-331-3p and NLRP6 was determined. To ensure a successful modeling, the brain slices and the cerebral blood flow were observed. The hematomas could be observed in the basal ganglia (Figure 5(a)), and the blood flow around the bleeding lesions was significantly reduced compared with the control group (Figure 5(b)). It was found that miR-331-3p in hemorrhagic brain tissues significantly decreased, when compared to the control group (Figure 6(a)). The real-time quantitative PCR, western blot, and immunohistochemistry revealed that NLRP6 exhibited an increase in tendency from the mRNA level to the protein level (Figures 6(b)–6(e)).

### 3.6. miR-331-3p Aggravates Inflammation Response and Alleviates the Recovery of Neurological Deficits in the ICH Mouse Model

**Figure 7:**
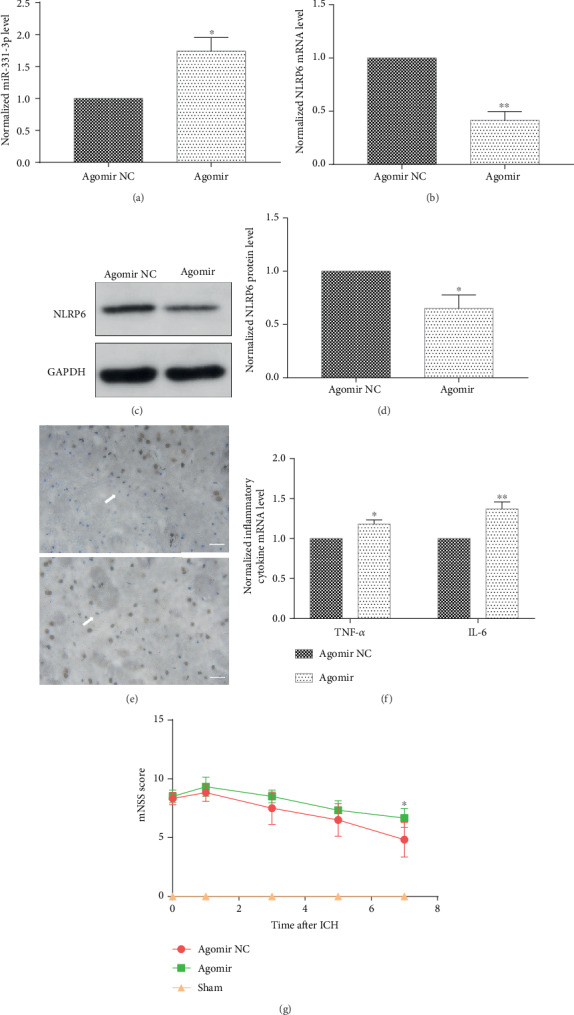


The rescued effect of miR-331-3p was further explored. Agomirs are microRNA mimics for animals (Figure 7(a)). Agomir-331-3p was injected into mice to imitate the miRNA-331-3p functions. After the intervention process, it was found that the activation of miR-331-3p led to the downregulation of NLRP6 around the hematoma tissue (Figures 7(b)–7(e)), and this was accompanied by an increase in inflammatory response (Figure 7(f)). The modified neurological severity score (mNSS) was used to evaluate the recovery of neurological deficits in mice. The scores from the first day to the seventh day after surgery for ICH mice were recorded, and it was found that the neurological function of mice injected with miR-331-3p mimics was less restored (Figure 7(g)).

## 4. Discussion

At present, the treatment of ICH mainly focuses on hematoma aspiration, including minimally invasive hematoma evacuation, neuroendoscopic surgery, and nonsurgical drug treatment. However, it remains hard to completely reverse the damage caused by cerebral hemorrhage to the nervous system, and this cannot significantly improve clinical outcomes [16]. These therapeutic constraints are mainly due to the ambiguity of the mechanism of injury after ICH.

Inflammatory damage is an indispensable factor that affects the prognosis of cerebral hemorrhage. Microglial expression is most active in 1-3 days after ICH, and the activation can last up to 28 days [3, 17, 18]. Inflammatory reactions can be observed in a short period of time after ICH in the mouse model. Wang et al. reported that neutrophil infiltration around the hematoma could be observed in as early as four hours after cerebral hemorrhage. Furthermore, a large amount of neutrophil around the hematoma could be detected at 1-3 days after cerebral hemorrhage [2].

After cerebral hemorrhage, microglia quickly respond to the changes of the internal environment. On the one hand, this activates microglial phagocytose necrotic cells, removes cell debris, reduces cell damage, and promotes tissue repair. On the other hand, the activated microglia secrete proinflammatory factors, such as TNF-*α* and IL-6. The activation of proinflammatory and neurotoxic factors recruit inflammatory cells, which release inflammatory cytokines, activate downstream signaling pathways, causing inflammatory cascades, and eventually lead to tissue edema aggravation, blood-brain barrier destruction, and nerve cell apoptosis. Hence, microglia need to be precisely regulated. In the present study, TNF-*α* and IL-6 were chosen as typical inflammatory cytokine makers. TNF-*α* and IL-6 are the proinflammatory factors commonly found in inflammatory reactions, which react to inflammatory response in the early stage and persist for a long time.

Hemin is a toxic product of hemoglobin degradation after cerebral hemorrhage. As a lipophilic oxidant, hemin can extremely damage various nerve cells in the brain [19, 20]. It has been reported that hemin can cause irreversible damage to neurons at a concentration of 4 *μ*mol/L [15]. In the present study, hemin was chosen to induce microglial activation *via* NLRP6. The concentration of hemin was chosen to be at 60 *μ*mol/L, which induced a significant inflammatory response, but did not cause excessive cell apoptosis.

The NLRP family has been considered as a high-profile protein family in recent years. It is mainly involved in immune response and plays an important role in apoptosis, inflammation, and oncology. Glorioso et al. reported that the D11S1318 and D11S1346 loci were correlated to hypertension susceptibility. The D11S1318 locus is a NLRP6/angiotensin vasopressin receptor [21], and it was suggested that NLRP6 might play a role in preventing the onset of ICH by reducing the genetic susceptibility of essential hypertension [22]. The transcription factor binding analysis revealed that peroxisome proliferator-activated receptor gamma (PPAR-*γ*) and retinoid X receptor-*α*, as well as the ovalbumin upstream promoter transcription factor 1 nuclear transcription factor binding site, were overexpressed in the 1,000 bp region upstream of the transcription initiation site of the NLRP6 gene [23]. In the previous study conducted by the investigators, it was found that rosiglitazone, which is a PPAR-*γ* agonist, increased the expression of PPAR-*γ* around the hematoma in cerebral hemorrhage and improved the neurological function in animal models, thereby providing evidence that NLRP6 might play a role in cerebral hemorrhage [24, 25].

miRNA has only been developed for approximately 20 years, and its biological field has been greatly expanded. Understanding the role of miRNAs in biological development and disease, particularly in cancer, has made miRNAs an attractive tool or target for novel therapeutic approaches [26–28]. miRNAs, as tumor suppressors or oncogenes (oncomiRs), miRNA mimics, and molecules that target miRNA (antimiRs), have promising prospects in preclinical applications [29, 30]. The mimic of miR-34 has been applied to preclinical stages for suppressing lung cancer, pancreatic cancer, and prostate cancer [31–33]. miR-122 upregulates the replication of the hepatitis C virus RNA genome [34]. The inhibitor of miR-122 has become a hotspot for the treatment of hepatitis C [35], and a phase II clinical trial has been conducted. Central nervous system damage, especially stroke, is a disease with a high rate of clinical disability.

There are few studies on miR-331, which have mainly focused on cancer research. miR-331-3p is a member of the miR-331 family, which is located on the 12q22n chromosome. miR-331 regulates cell cycle progression in human gastric cancer [36, 37] and promotes the development and progression of glioblastoma by increasing cell proliferation and clonal growth [38]. miR-331-3p has been proven to be a tumor regulator in prostate cancer, which acts as a mediator of ErbB-2 expression and PI3K/AKT signaling [39, 40]. Furthermore, miR-331-3p regulates tumor growth, predicts the prognosis, and manages the expression of many RNA and growth factor receptors in adult hemangioma [41]. At the same time, in the proliferation and migration of lung cancer [42, 43] and lymphocytic leukemia [44], miR-331-3p has been observed to be abnormally expressed in pathophysiological changes, and its function differs due to the differences in tissue structure. The present study was the first to reveal that miR-331-3p may play a role in the inflammatory response following cerebral hemorrhage, which could lay a foundation for the study of miR-331-3p in the field of stroke. miRNAs could be considered as a new therapeutic strategy for neuroprotection. Due to the nonrenewable nature of nerve cells, the treatment of central nervous system diseases often has limitations. The special role of miRNA in disease progression shows that attention should be given to the treatment of central nervous system diseases. One of the key points in the present use of miRNA mimics and inhibitors is to determine how to efficiently and accurately deliver miRNAs to the target sites. In order to achieve this goal, there is a need to design a suitable delivery tool to transfer these miRNAs to the targeted area and ensure that these miRNAs cannot be cleared during the immunosurveillance process.

In conclusion, from the *in vitro* level to the *in vivo* level, the present study is the first to propose that miR-331-3p regulates the inflammatory response after cerebral hemorrhage by negatively regulating the expression of NLRP6. The downregulation of miR-331-3p increased the expression of NLRP6, alleviated the inflammatory response, and promoted the neurological function recovery of mice after ICH.

## Data Availability

The data used to support the findings of this study are included within the article.
